# A High-Performance Liquid Chromatography Assay Method for the Determination of Lidocaine in Human Serum

**DOI:** 10.3390/pharmaceutics9040052

**Published:** 2017-11-18

**Authors:** Hamdah M. Al Nebaihi, Matthew Primrose, James S. Green, Dion R. Brocks

**Affiliations:** 1 Faculty of Pharmacy and Pharmaceutical Sciences, University of Alberta, Edmonton, AB T6G 2E1, Canada; alnebaih@ualberta.ca; 2 Department of Anesthesiology and Pain Medicine, Faculty of Medicine and Dentistry, University of Alberta, Edmonton, AB T6G 2B7, Canada; mprimros@ualberta.ca (M.P.); jgreen2@ualberta.ca (J.S.G.)

**Keywords:** lidocaine, pharmacokinetics, ultraviolet detection

## Abstract

Here we report on the development of a selective and sensitive high-performance liquid chromatographic method for the determination of lidocaine in human serum. The extraction of lidocaine and procainamide (internal standard) from serum (0.25 mL) was achieved using diethyl ether under alkaline conditions. After liquid–liquid extraction, the separation of analytes was accomplished using reverse phase extraction. The mobile phase, a combination of acetonitrile and monobasic potassium phosphate, was pumped isocratically through a C18 analytical column. The ultraviolet (UV) wavelength was at 277 nm for the internal standard, and subsequently changed to 210 for lidocaine. The assay exhibited excellent linearity (*r*^2^ > 0.999) in peak response over the concentration ranges of 50–5000 ng/mL lidocaine HCl in human serum. The mean absolute recoveries for 50 and 1000 ng/mL lidocaine HCl in serum using the present extraction procedure were 93.9 and 80.42%, respectively. The intra- and inter-day coefficients of variation in the serum were <15% at the lowest, and <12% at other concentrations, and the percent error values were less than 9%. The method displayed a high caliber of sensitivity and selectivity for monitoring therapeutic concentrations of lidocaine in human serum.

## 1. Introduction

Lidocaine [also known as 2-(diethylamino)-*N*-(2,6-dimethylphenyl) acetamide] is commonly used as a local anesthetic and antiarrhythmic drug [1]. It can also be used for the management of extensive pain via either central or peripheral administration [2]. For these reasons, lidocaine is considered an essential medication for a wide array of clinical conditions [3]. Lidocaine is not a new medication, and hence many methods are available for its quantitation in biological specimens. The available methods are wide ranging, as is evident from the summary provided in Table 1. The most commonly used approach is to use reverse phase high performance liquid chromatography for separation, and ultraviolet (UV) absorbance as the detection method. This is not surprising considering that lidocaine by itself has poor fluorescence properties, and UV detectors are commonly available in laboratories.

Recently, our involvement in a clinical study necessitated that we measure lidocaine from human serum. Most of the previously available methods were based on volumes of 0.5 to 1 mL of specimen. In order to ensure that we could assay all of the samples available, we sought to develop a method that would use only 0.25 mL of specimen. Here we report on an alternative analytical method for lidocaine that was sufficiently sensitive to measure the drug in the serum using a volume of 0.25 mL.

## 2. Materials and Methods

**Figure 1:**
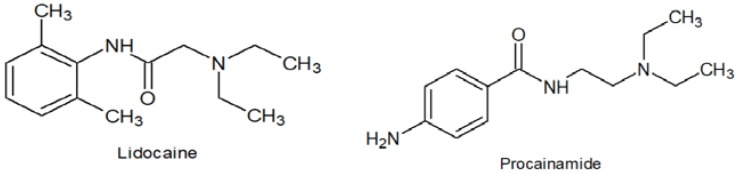
Chemical structure of lidocaine and procainamide, the internal standard.

Lidocaine HCl (0.4% in 5% dextrose for injection) USP (Baxter Healthcare Corporation, Deerfield, IL, USA) was utilized as a source of lidocaine (Figure 1). Procainamide HCl was obtained from Sigma-Aldrich (St. Louis, MO, USA) (Figure 1). Ethyl ether, acetonitrile, water (all HPLC grade), triethylamine, potassium phosphate (monobasic) and sulfuric acid were purchased from Caledon Laboratories Ltd. (Georgetown, ON, Canada).

### 2.1. Instrumentation and Chromatographic Conditions

The chromatographic system consisted of a Waters (Milford, MA, USA) 600 E multi-solvent delivery system pump, an autosampler with a variable injection valve (Waters 717), and a UV–visible tunable absorbance detector (Waters 486). The chromatograms were recorded using EZStart software (Scientific Software, Pleasanton, CA, USA) in a Windows-based computer system for data collection and processing. The chromatographic separation of lidocaine and procainamide (internal standard) were achieved using a 150 × 4.6 mm i.d., 3.5 μm particle size Alltima C18-column (Alltech, Deerfield, IL, USA) attached to a pre-guard column (Grace Alltech All-Guard™ Guard Cartridges, 7.5 mm 5 µm, Deerfield, IL, USA). The mobile phase consisted of a mixture of acetonitrile and phosphate solution (25 mM KH_2_PO_4_-3 mM sulfuric acid-3.6 mM triethylamine) in a ratio of 12:88 (*v*/*v*). The mobile phase was degassed prior to use by filtering it under vacuum pressure through a 0.45 μm pore size nylon filter, then pumped at a flow rate of 0.9 mL/min at room temperature.

Immediately after injection, the UV detection wavelength was set at 277 nm (the UV_max_ for procainamide). At 4 min post-injection, the UV detector was programmed to switch to a wavelength of 210 nm (the UV_max_ for lidocaine). The total analytical run time was <13 min.

### 2.2. Standard and Stock Solutions

The stock drug solution was 0.4% lidocaine HCl in 5% dextrose for injection. The working standard solutions were prepared daily from the stock solution by serial dilution with HPLC grade water to provide final concentrations of 40, 4 and 0.4 μg/mL lidocaine. The internal standard (IS) stock solution was prepared by dissolving 17 mg of procainamide HCl in 100 mL of water. All stock solutions were refrigerated between use. For the construction of a standard curve, samples of (0.25 mL) were prepared by adding lidocaine HCl equivalent to 50, 125, 250, 500, 1000, 2000, 2500 and 5000 ng/mL.

### 2.3. Extraction Procedure

A one-step liquid–liquid extraction step was used to extract lidocaine from human serum. After adding 50 μL volume of the IS stock solution and 200 μL of 1 M NaOH to 0.25 mL of human serum, 3 mL of diethyl ether was added. This was followed by vortex mixing of tubes for 30 s and centrifugation at 3000 *g* for 3 min. The organic solvent layer was pipetted to new tubes, then evaporated to dryness in vacuo. The dried residues were reconstituted using 150 μL of HPLC grade water with up to 75 μL being injected into the chromatographic system.

### 2.4. Recovery

Recovery was determined with lidocaine HCl concentrations of 50 and 1000 ng/mL and with an IS concentration of 0.17 mg/mL in human serum. The extraction efficiency was determined using five replicates of each concentration and comparing the extracted peak heights of analyte in the extracted samples to the peak heights of the same amounts of analyte directly injected into the HPLC without extraction.

### 2.5. Calibration, Accuracy and Validation

The assay was validated, generally using the guidelines published by the EMA [18]. The calibration curves were quantified by using peak height ratios of lidocaine HCl (concentration range from 50 to 5000 ng/mL) to IS versus the nominal lidocaine concentration. Intra-day validation was assessed at four different concentrations of lidocaine HCl (50, 250, 500 and 2000 ng/mL) per day in five replicates. This step was repeated on three separate days for determination of inter-day validation. For each daily run, an independent set of calibration curves samples was prepared. Accuracy and precision were assessed using the mean intra- and inter-day percentage error and percent coefficient of variation (CV%), respectively. Calibration curves were weighted by a factor of concentration^−2^ due to the wide range of concentrations (50–5000) used in the calibration curves.

Intraday, accuracy and precision of the assay were determined using a range of concentrations of lidocaine HCl. The selected concentrations were 50, 250, 500 and 2000 ng/mL of lidocaine HCl in human serum. Each concentration had five replicates. To permit the assessment of interday accuracy and precision in human serum, the assay was repeated on three separate days. For each daily run, a set of calibration samples separate from the validation samples were prepared to permit the quantification of the peak height ratios of lidocaine to IS. Precision was assessed by percentage coefficient of variation (CV%), while accuracy was represented by determining the mean intra- or inter-day percentage error.

### 2.6. Applicability

For the standard curve and validation samples, the serum was obtained from two healthy individuals on two separate occasions. For two of the samplings, each individual was asked to refrain from ingesting any caffeine-laden foods or drinks for 24 h before sampling.

The method was employed to determine the lidocaine concentration in human serum from one surgical patient after the injection of lidocaine as a reservoir within the rectus sheath (200 mg single dose). The patient provided written consent, and the study was approved by the University of Alberta Health Research Ethics Board. After the injection of the dose, blood samples were drawn serially into serum collection tubes until 24 h from the time of dosing. The blood samples were left for 30 min to clot, then centrifuged for 10 min. The serum was then separated and frozen at −20 °C until assayed.

## 3. Results

**Figure 2:**
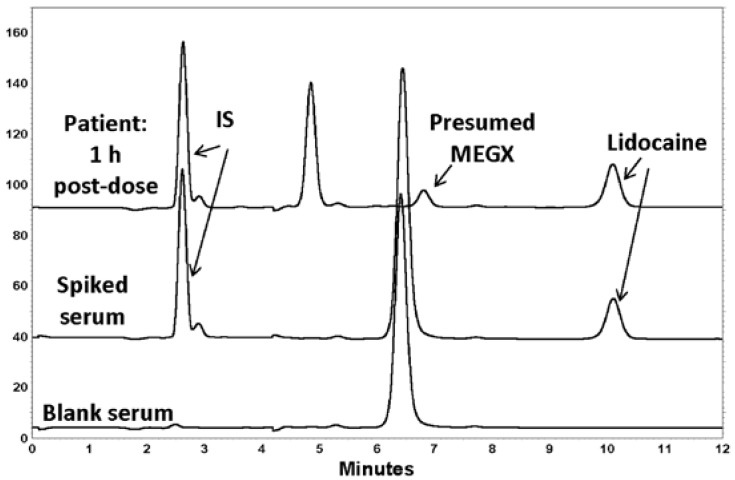
HPLC-UV chromatograms of blank (drug-free) serum from a healthy volunteer, volunteer serum spiked with 1000 ng/mL of lidocaine, and serum obtained 1 h after a 200 mg injection of lidocaine. The peak in the patient sample appearing at 6.8 min in the patient sample was presumed to be the lidocaine metabolite, monoethylglycinexylidide (MEGX). The peak at ~6.4 min was presumed to be from food components ingested by the volunteers who were not fasted.

The chromatographic retention times were ~10 min for lidocaine and 2.5 min for IS (Figure 2). The chromatography displayed symmetrical peak shapes and high specificity, with a baseline resolution of IS and lidocaine. For both analytes, there was an absence of interference from endogenous components in serum (Figure 2). It was of note that the chromatograms from the serum of the healthy volunteers used in the assay development had a significant peak present that eluted at about 6.4 min, which was not seen in the patient serum sample obtained at 10 min. However, this did not interfere with the analysis of lidocaine, nor the internal standard.

The average recoveries were 97.7 ± 21% and 81.0 ± 2.4% for 50 and 1000 ng/mL lidocaine HCl in serum, respectively. The average extraction recoveries for IS at the concentration used in the assay was 55.4 ± 4.1%. There were excellent linear relationships (>0.9995) noted between the peak height ratios and lidocaine HCl concentrations over ranges of 50–5000 ng/mL serum.

From regression analysis of the concentration vs. peak height ratios of lidocaine to IS, the observed average slopes and intercepts were 0.00024 and 0.0011, respectively. The mean correlation coefficient of regression (*r*^2^) for the serum standard curves were 0.9995. The CV of intra-and inter-day assessments were less than 15% (Table 2). The mean inter-day error in human serum was less than 9% (Table 2).

The validation assessments revealed that the assay was precise and possessed low bias. Overall, all intraday measures for mean error and CV% of the lowest concentration were less than 15%, and for the other concentrations only one fell above 10% (day three of the 216 ng/mL concentration). Except for the lowest concentration, all of the interday measures of CV% were less than 10%. All interday measures for mean error were less than 10%.

When tested for applicability in the patient samples, lidocaine serum concentrations could be measured for the full 24 h period following administration of the lidocaine dose. The observed maximal concentration was 1644 ng/mL, which occurred at 0.5 h post-dose.

The assay was reproducible and relatively fast in terms of preparation. To assay 30 samples, from the time of adding components to serum to completion of the evaporation of extracted solvent, took less than 40 min.

## 4. Discussion

The HPLC method described here represents an accurate and precise avenue to determine the concentration of lidocaine in 0.25 mL volumes of human serum. The volumes of serum required are at the lower end of those volumes used by other analytical methods (Table 1). Additionally, the reported validated lower limits of quantitation are frequently greater than 50 ng/mL (Table 1). The lowest validated quantifiable concentration was found in the literature to be 10 ng/mL, though a serum volume of 1 mL was required [8]. Sintov et al. reported that the validated lower limit of quantification by using fluorescence detector was 25 ng/mL, which also required larger volumes of specimen up to 1 mL and derivatization following the extraction process [12], thereby adding elements of expense and time into sample preparation. The lower limit of quantitation (LLQ) in the current method is comparable to 50 ng/m by the GC-MS method [4].

Alkaline conditions were used to force lidocaine into its free base form, which promotes its extraction into a suitable organic solvent [12]. For the extraction of lidocaine from biological matrices, different solvents have been used in HPLC methods. These include an ethyl acetate/hexane/methanol mixture [14], dichloromethane [5], ethyl acetate [7,11], and a mixture of diethyl ether/dichloromethane [8]. Qin et al. had previously used diethyl ether as an extraction solvent for local anesthetics in human plasma, including procaine, ropivacaine, tetracaine, bupivacaine, including lidocaine [9]. The extraction of lidocaine from plasma in their hands was virtually complete, similar to what we had found. For this reason, we adopted the extraction method of Qin for use in the currently described assay method. Qin et al. had used carbamazepine as their internal standard. Due to its late elution time compared to procainamide [9], we chose to use procainamide instead. Although its recovery was less than that of lidocaine, it nevertheless extracted very consistently and provided excellent standard curves and validation indices. Procainamide would be an unlikely interfering substance in a patient as while it is chemically similar to lidocaine, it is not a popular antiarrhythmic currently, nor is it used as an anesthetic agent. The disadvantage of procainamide is that it requires that the UV detector possess the ability to program a change in wavelength after the elution of the internal standard.

In terms of mobile phase composition, acetonitrile and phosphate solution are the most common components in most of the reported studies (Table 1). Only Tam et al. and Qin et al. supplemented the mobile phase with 0.15% and 0.16% of triethylamine (TEA), respectively [7,9], while Chen et al. supplied the mobile phase with 1% diethylamine [6]. In the current method, 0.15% of triethylamine was added as it improved the peak symmetries and reduced the peak tailing of the compounds of interest, as has been noted for other compounds [19,20]. From the literature, the retention time for the compound of interest ranged from 3.6 to less than 13 min (Table 1).

**Figure 3:**
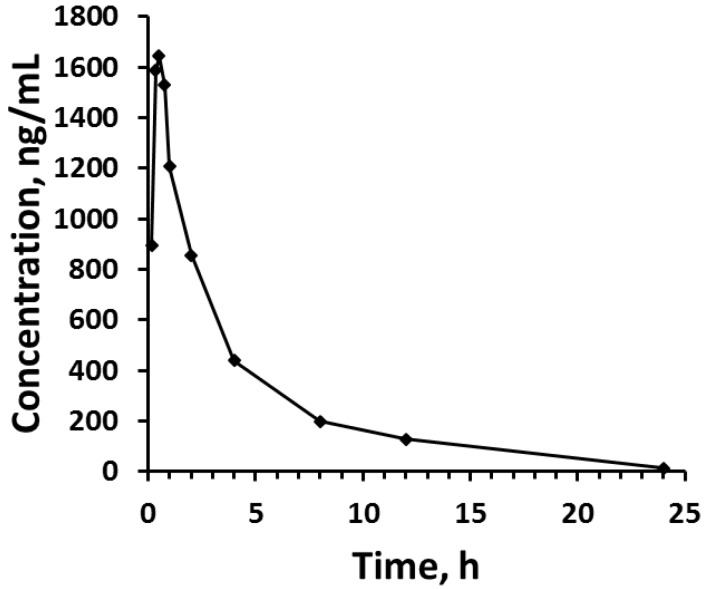
Serum lidocaine concentration vs. time profiles after rectus sheath injection of 200 mg lidocaine HCl to the patient volunteer.

The measured serum concentrations in the patient were in accordance with measures of plasma concentrations observed previously in patients administered the same dose of lidocaine HCl as depot parenteral injections [21]. The presence of a peak at 6.4 min was noticed in HPLC-UV chromatograms of blank (drug-free) human serum. Unfortunately, the appearance of this peak in the blank serum (with or without a caffeine-free period) virtually coincided with the elution of monoethylglycylxylidide (MEGX), a major metabolite of lidocaine [22]. In the first sample taken from the patient 10 min after the dose, although lidocaine was present, there was no peak observed eluting between 6 and 7 min. In later timed samples, such as 1 h after dosing (Figure 3) a peak virtually coinciding with MEGX was apparent. However, because in the volunteer serum, a large, mostly interfering peak was present, it was not possible to quantify the MEGX concentrations in the serum of the patient using the chromatographic conditions optimal for lidocaine elution. The patient differed from those subjects contributing volunteer serum in that the patients were required to fast before the surgery. Hence, it is possible that the peak in the healthy serum was from a food component, as none of the healthy volunteers had consumed any medications before contributing the blood for serum. The assay of metabolite was not a primary aim of this study, so we did not further pursue assay work as it would have meant a longer analytical run time for lidocaine, our primary analyte of interest. We did check for the elution of caffeine, and indeed it eluted at the same time as the large interfering peak in the serum of the volunteers who drank caffeine, but it was still present even after a 24 h caffeine fast. Therefore, it most likely derived from some other component of food. The patient also received other medications during surgery, and we believe that one of these may have been represented by the peak at 5.8 min (Figure 3), as it was not also seen in the volunteer serum, none of whom ingested any other drugs orally.

## 5. Conclusions

The method demonstrated high calibers of sensitivity and selectivity for monitoring concentrations of lidocaine in human serum. The method was uncomplicated in terms of sample preparation and extraction. It was precise and accurate and was shown to have utility as a means to measure lidocaine concentration as part of a pharmacokinetic study.

## Figures and Tables

**Table 1:** Comparisons of some published methods for assaying lidocaine in human matrices.

Volume of Specimen (mL)	Validated LLQ (ng/mL)	Type of Human Matrix	Sample Preparation Method	Analytical Column	Detection Method	References
0.2	50	Plasma	SPE	DB-1	GC–MS	[4]
0.5	200	Serum	LLE	C8	UV	[5]
0.1	680	Plasma	Protein ppt	C18	UV	[6]
0.5	200	Plasma	LLE	C18	UV	[7]
1	10	Serum	LLE	C18	UV	[8]
0.5	50	Plasma	LLE	C18	UV	[9]
0.1	400	Plasma	Protein ppt	C18	UV	[10]
0.5	100	Plasma	LLE	Phenyl	UV	[11]
0.5–1	25	Plasma	LLE	C18	Fluorescence of derivative	[12]
0.25	NS	Plasma	NS	C18	UV	[13]
1	1000	Plasma	LLE	C18	UV	[14]
0.01	200	Plasma	Protein ppt	C18	LC-MS/MS	[15]
1	0.2	Plasma	LLE	C18	LC-MS/MS	[16]
1	20	Serum	None	C18	UV	[17]
0.25	43	Serum	LLE	C18	UV	Current method

**Table 2:** Validation data for the assay of lidocaine in human serum, *n* = 5.

Nominal Concentration of Lidocaine *, ng/mL	Intraday			Interday		
	Mean ± SD ng/mL (CV%)			Mean ± SD ng/mL	CV%	Error%
43.3	45.9 ± 4.54 (9.90)	38.5 ± 5.37 (14.0)	40.8 ± 6.05 (14.8)	41.7 ± 5.32	12.9	−3.57
216	2158 ± 11.5 (5.35)	220 ± 9.60 (4.35)	226 ± 26.6 (11.8)	221 ± 15.9	7.16	1.99
433	379 ± 28.7 (7.56)	395 ± 25.6 (6.46)	407 ± 38.5 (9.47)	394 ± 30.9	7.83	−8.97
1731	1489 ± 60.5 (4.06)	1704 ± 43.3 (2.53)	1635 ± 47.1 (2.87)	1609 ± 50.3	3.16	−7.02

* To convert to lidocaine HCl salt, divide by 0.865.

## Abbreviations

CV%	Coefficient of variation expressed as percent
LLQ	Lower limit of quantitation
SPE	Solid phase extraction
GC-MS	Gas chromatography-mass spectrometry
ppt	Precipitation
IS	Internal standard
LLE	Liquid-liquid extraction
UV	Ultraviolet detection
NS	Not specified or unclear
